# GRAF1 Acts as a Downstream Mediator of Parkin to Regulate Mitophagy in Cardiomyocytes

**DOI:** 10.3390/cells13050448

**Published:** 2024-03-04

**Authors:** Qiang Zhu, Matthew E. Combs, Dawn E. Bowles, Ryan T. Gross, Michelle Mendiola Pla, Christopher P. Mack, Joan M. Taylor

**Affiliations:** 1 Department of Pathology and Laboratory Medicine, University of North Carolina, Chapel Hill, NC 27599, USA; qiangzhu@email.unc.edu (Q.Z.); matthew_combs@med.unc.edu (M.E.C.); cmack@med.unc.edu (C.P.M.); 2 Division of Surgical Sciences, Duke University Medical Center, Durham, NC 27710, USA; dawn.bowles@duke.edu (D.E.B.); rtg13@duke.edu (R.T.G.); michelle.mendiola.pla@duke.edu (M.M.P.); 3 McAllister Heart Institute, University of North Carolina, Chapel Hill, NC 27599, USA

**Keywords:** GRAF1, PINK1-Parkin pathway, mitophagy, cardiomyocytes, metabolism

## Abstract

Cardiomyocytes rely on proper mitochondrial homeostasis to maintain contractility and achieve optimal cardiac performance. Mitochondrial homeostasis is controlled by mitochondrial fission, fusion, and mitochondrial autophagy (mitophagy). Mitophagy plays a particularly important role in promoting the degradation of dysfunctional mitochondria in terminally differentiated cells. However, the precise mechanisms by which this is achieved in cardiomyocytes remain opaque. Our study identifies GRAF1 as an important mediator in PINK1-Parkin pathway-dependent mitophagy. Depletion of GRAF1 (*Arhgap26*) in cardiomyocytes results in actin remodeling defects, suboptimal mitochondria clustering, and clearance. Mechanistically, GRAF1 promotes Parkin-LC3 complex formation and directs autophagosomes to damaged mitochondria. Herein, we found that these functions are regulated, at least in part, by the direct binding of GRAF1 to phosphoinositides (PI(3)P, PI(4)P, and PI(5)P) on autophagosomes. In addition, PINK1-dependent phosphorylation of Parkin promotes Parkin-GRAF1-LC3 complex formation, and PINK1-dependent phosphorylation of GRAF1 (on S668 and S671) facilitates the clustering and clearance of mitochondria. Herein, we developed new phosphor-specific antibodies to these sites and showed that these post-translational modifications are differentially modified in human hypertrophic cardiomyopathy and dilated cardiomyopathy. Furthermore, our metabolic studies using serum collected from isoproterenol-treated WT and GRAF1^CKO^ mice revealed defects in mitophagy-dependent cardiomyocyte fuel flexibility that have widespread impacts on systemic metabolism. In summary, our study reveals that GRAF1 co-regulates actin and membrane dynamics to promote cardiomyocyte mitophagy and that dysregulation of GRAF1 post-translational modifications may underlie cardiac disease pathogenesis.

## 1. Introduction

Mitochondria play vital roles in cellular functions, serving as the central hub for cellular metabolism by providing energy through oxidative phosphorylation. Cardiomyocytes, being highly metabolically active and rich in mitochondria, rely heavily on mitochondrial ATP production for continuous contraction. In addition to their role in ATP generation through electron transport activity, mitochondria also yield reactive oxygen species (ROS), providing substrates for signaling reactions and essential metabolites for anabolism. However, mitochondria also contribute to cell death processes like apoptosis and necrosis [1,2]. Disruption of mitochondrial function can jeopardize cellular redox balance, impair calcium homeostasis, and lead to metabolic disorders, ultimately resulting in cell death [3,4,5,6]. Thus, maintaining mitochondrial quality is crucial for cell homeostasis, especially in long-lived cells like cardiomyocytes [7]. Mitophagy, a process that involves the selective engulfment of damaged mitochondria into autophagosomes followed by degradation in lysosomes, plays a critical role in eliminating dysfunctional mitochondria and safeguarding cellular health [8,9,10].

The PINK1-Parkin pathway is a critical signal transduction cascade for mitophagy. Under normal conditions, the serine/threonine kinase PTEN-induced putative kinase 1 (PINK1) is imported into healthy mitochondria by the translocase of the outer membrane and translocase of the inner membrane (TOM and TIM) complexes. Once inside the mitochondria, PINK1 is cleaved by the matrix processing peptidase (MPP) and the inner membrane protease presenilin-associated rhomboid-like protease (PARL) [11,12]. However, in the presence of damaged mitochondria, the processes of mitochondrial protein translocation and processing are compromised, leading to the accumulation and stabilization of PINK1 on the mitochondrial outer membrane (MOM). Activated PINK1 phosphorylates Ser65 of pre-existing ubiquitin at the mitochondrial surface, which results in the recruitment and activation of Parkin [13,14,15]. Fully activated Parkin then initiates conjugation of ubiquitin to various substrates on the MOM, providing new ubiquitination sites to be phosphorylated by PINK1, thereby establishing a feedforward amplification loop [16,17,18,19,20,21,22]. Adaptor proteins, such as P62, NBR1, NDP52, OPTN, and TAX1BP1, possess both ubiquitin-binding domains and microtubule-associated proteins 1A/1B light chain 3 (MAP1LC3, here referred to as LC3) interacting region (LIR) motifs [23,24,25]. These features enable them to facilitate the interaction between ubiquitinated proteins on MOM and autophagosomes, thereby guiding autophagosomes to damaged mitochondria and ensuring the selective removal of dysfunctional mitochondria [26,27,28,29]. Dysregulation in this process is implicated in diseases such as Parkinson’s disease and heart failure [10,30,31,32,33].

While the PINK1-Parkin pathway is recognized as a critical signal transduction cascade for mitophagy and is particularly crucial for long-lived and highly differentiated cells such as cardiomyocytes and neurons, it is essential to acknowledge that the precise mechanism and regulatory pathway of this process in these specialized cells remain insufficiently understood. Consequently, exploring the intricate mechanisms governing mitophagy and modulating its activity holds promise as a potential therapeutic strategy for treating or preventing cardiac diseases.

The GRAF family is characterized by multiple domains, including BAR (Bin/Amphiphysin/Rvs), PH (pleckstrin homology), GAP, and SH3 (Src homology 3 domains). This distinctive structural arrangement positions them to cooperatively regulate actin and membrane dynamics. GRAF1 limits RhoA-dependent actin polymerization via its GAP domain. The N-terminal BAR and PH domains induce membrane curvature and determine binding specificity, and the C-terminal SH3 domain mediates protein-protein interactions with partners that control endocytic recycling, adhesion, and/or branched actin remodeling (e.g., dynamin, focal adhesion kinase, Abi2) [34,35,36,37,38]. Interestingly, we recently reported that GRAF1 plays a critical role in regulating PINK1/Parkin-dependent mitophagy by regulating precise spatial and temporal control of actin remodeling [39]. Notably, we demonstrated using a Hela cell model that GRAF1 plays a role in facilitating F-actin de-polymerization induced by mitochondrial poisons. This process results in the release of mitochondria from F-actin cables, promoting mitochondrial trafficking and generating G-actin monomers for subsequent polymerization. Additionally, GRAF1 is recruited to the outer mitochondrial membrane (OMM) in response to damaged mitochondria and undergoes phosphorylation by PINK1 and/or PINK1-dependent kinases. PINK1-dependent phosphorylation of GRAF1 relieves SH3 domain auto-inhibition, drives SH3-mediated interactions with ABI2, and promotes mitochondrial recruitment of an ABI2/WAVE2/Arp2/3 branched actin complex to facilitate mitochondrial clustering and clearance. Our findings that GRAF1 also forms a complex with Parkin and LC3 add another layer of specificity to this process. Herein, we defined the molecular interactions that drive this association, explore the relationship of specific post-translational modifications of GRAF1 in human heart conditions, and explore the consequences of cardiac-restricted GRAF1 depletion on whole body homeostasis.

## 2. Materials and Methods

### 2.1. Mouse Model and ISO Treatment

In accordance with the methodology detailed in the provided reference [39], GRAF1flox/+ mice were initially generated. Subsequently, crossbreeding was carried out between GRAF1flox/+ mice and hemizygous αMHC-MerCreMer (MCM) mice, resulting in the production of αMHC-MCM/+: GRAF1flox/+ mice. This newly established lineage was further mated with hemizygous αMHC-MCM mice, giving rise to αMHC-MCM/MCM: GRAF1flox/+ mice. Male αMHC-MCM/MCM: GRAF1flox/+ mice were then bred with female GRAF1flox/+ mice to generate hemizygous αMHC-MCM mice as controls and αMHC-MCM/+: GRAF1flox/flox mice as tamoxifen-inducible cardiac-restricted knockout mice. For tamoxifen induction, male αMHC-MerCreMer/+ and αMHC-MerCreMer/+: GRAF1flox/flox mice (2–3 months old) received a single intraperitoneal injection of 40 mg/kg tamoxifen (Sigma, St. Louis, MO, USA, T5648-1G) dissolved in sterile peanut oil. In the chronic β-adrenergic stimulation model, mice were subjected to a 14-day treatment with Isoproterenol (ISO) (Sigma, I6504-1G) at a dosage of 15 mg/kg/day, starting 7 days post tamoxifen injection. Subcutaneously implanted osmotic pumps (model 2002, Alzet, Cupertino, CA, USA) were utilized for administering ISO. All mice were housed in pathogen-free facilities, adhering to a 12-h light/dark cycle, and had unrestricted access to food and water. Animal procedures were conducted following the approved protocol of the University of North Carolina (Chapel Hill, NC, USA) Institutional Animal Care and Use Committee, in compliance with the standards outlined in the guide for the Care and Use of Laboratory Animals.

### 2.2. Cell Culture and Transfection

Hela cells expressing YFP-Parkin (Hela/YFP-Parkin) were generously provided by Dr. Richard Youle at the National Institutes of Health (NIH). COS7 cells and Hela cells were cultured in Dulbecco’s modified Eagle’s medium (DMEM) (Gibco, Waltham, MA, USA, 11965092) supplemented with 10% fetal bovine serum (FBS) (Sigma, St. Louis, MO, USA, F4135) and 100 units penicillin per 100 µg streptomycin (final concentration).

Plasmids containing the desired cDNA variants were generated using either site-directed mutagenesis PCR (Agilent, Santa Clara, CA, USA, 200515) or Gibson Cloning (New England BioLabs, Ipswich, MA, USA, E2611L), following the manufacturer’s protocols. These plasmids were transfected into Hela or COS7 cells using Lipofectamine™ 2000 Transfection Reagent (ThermoFisher, Waltham, MA, USA, 11668027) for 20 h. Mitophagy induction was achieved by treating transfected cells with 2.5 μM oligomycin (Sigma, 75351-5 mg) and 250 nM antimycin A (Sigma, A8674-100 mg) for 6 h. Hela cells were transfected with target siRNA using Lipofectamine™ RNAiMAX Transfection Reagent (ThermoFisher, 13778075) for 72 h.

Primary neonatal rat ventricle cardiomyocytes (NRVCMs) were isolated from 2 to 3-day-old Wistar rats following the Neonatal Cardiomyocyte Isolation System protocol (Worthington Biochemical Corporation, Lakewood, NJ, USA, LK003300). NRVCMs were transfected with target siRNA using a Hiperfect transfection reagent (Qiagen, Germantown, MD, USA, 301707), with two rounds of transfection over 48-h intervals.

For drug treatments, Hela cells were exposed to either vehicle (DMSO) or 10 μM oligomycin and 5 μM antimycin A for short durations (up to 6 h) or with 2.5 μM oligomycin and 250 nM antimycin A for longer periods (16 or 20 h). Similarly, NRVCMs were treated with either vehicle (DMSO) or 5 μM oligomycin and 5 μM antimycin A for various indicated time periods.

Human PINK1 stealth siRNA (Catalog# HSS127945) and stealth negative control siRNA (Catalog# 12935110) were directly purchased from ThermoFisher. For siRNA transfection in NRVCMs and Hela cells, specific sequences targeting GRAF1 and GFP were used, as detailed below.

Stealth siRNA Transfection

NRVCMs:

GRAF1 stealth siRNA:

Forward: 5′-CGGAAGUUUGCAGAUUCCUUAAAUG-3′

Reverse: 5′-CAUUUAAGGAAUCUGCAAACUUCCG-3′

GFP stealth siRNA:

Forward: 5′-GGUGCGCUCCUGGACGUAGCC[dT][dT]-3′

Reverse: 5′-GGCUACGUCCAGGAGCGCACC[dT][dT]-3′

Hela Cells:

Stealth GRAF1 siRNA:

Forward: 5′-CAUUUCUAUGAAGUAUCCCUGGAAU-3′

Reverse: 5′-AUUCCAGGGAUACUUCAUAGAAAUG-3′.

### 2.3. Immunocytochemistry and Light Microscopy

Precision NO 1.5 H coverslips (Thorlabs, Newton, NJ, USA, CG15NH1) were coated with 5 μg/mL Fibronectin (R&D Systems, Minneapolis, MN, USA, 1030-FN-05M) overnight at 4 °C. Subsequently, NRVCMs were plated on the coverslips according to the specific experimental requirements. After relevant treatments, cells were rinsed in PBS and fixed with PBS-buffered 4% paraformaldehyde for 15 min at room temperature.

For staining of mitochondrial matrix proteins HSP60, cells were permeabilized with PBS containing 0.1% Triton X-100 at room temperature for 10 min. Alternatively, for staining of all other targets, cells were permeabilized with PBS containing 0.1% saponin for 15 min at room temperature. Following permeabilization, cells were blocked in PBS containing 10% goat serum for 1 h at room temperature. Slides were then subjected to a 1-h incubation with primary antibodies at room temperature, followed by a 1-h incubation with fluorophore-conjugated secondary antibodies. Finally, slides were mounted with ProLong Diamond Antifade Mountant (ThermoFisher, Waltham, MA, USA, P36970) overnight at room temperature.

The primary antibodies used were mouse anti-HSP60 (sc-13115, Santa Cruz, Dallas, TX, USA, 1:250) and rabbit anti-GRAF1 (homemade antibody, 1:250), mouse anti-LC3B (sc-398822, Santa Cruz, 1:100). Secondary antibodies included highly cross-adsorbed goat anti-mouse Alexa Fluor 488 (A-11001, ThermoFisher, 1:500), goat anti-rabbit Alexa Fluor 488 (A11008, ThermoFisher, 1:500), and donkey anti-rabbit Alexa Fluor 555 (A31570, ThermoFisher, 1:500). F-actin was stained with Phalloidin Alexa Fluor 555 (A34055, ThermoFisher, 1:100).

For confocal microscopy, imaging was acquired using a 63×/1.4 Plan Apo Oil objective on an LSM 700 confocal laser-scanning microscope (Zeiss, Oberkochen, Germany). Images for comparison were consistently acquired with the same settings, utilizing single-plane imaging. Adjustments for gamma, brightness, and contrast were uniformly applied using Fiji.

### 2.4. Bacterial Protein Expression, Purification, and Pulldown Assays

The full-length GRAF1b isoform and various GRAF1 domains were cloned into the pGEX-6P-1 vector and expressed as glutathione-s-transferase (GST) fusion proteins in BL21 *E. coli*. The expressed GST-fusion proteins were subsequently purified using Glutathione Sepharose 4B (Cytiva, Marlborough, MA, USA, 17075601). For GST pull-down assays, COS7 cells transfected with GFP-LC3 plasmids or neonatal rat ventricle cardiomyocytes (NRVCMs) were collected in lysis buffer (20 mM Tris HCl, 150 mM NaCl, 10% glycerol, 0.5% Triton X100, pH 7.4, with 1× HALT phosphatase and protease inhibitor cocktail). After a 15-min incubation on ice, the cell lysates were centrifuged at 20,000× *g* for 15 min. The supernatants were then incubated with GST-fusion protein-loaded beads for 1 h with end-over-end rotation at 4 °C. Beads underwent five washes with cell lysis buffer and were subsequently eluted in 2× sample loading buffer for Western blot analysis.

### 2.5. Co-Immunoprecipitations and Western Blot Analyses

For co-immunoprecipitation of target proteins from COS7 cell lysates, COS7 cells were transfected with specified plasmids, expressing the target proteins for 20 h. Subsequently, transfected COS7 cells underwent treatment with a combination of Oligomycin (2.5 μM) and Antimycin A (250 nM) for 6 h to induce mitophagy. To conduct co-immunoprecipitation of target proteins from NRVCMs, NRVCMs were treated with Oligomycin (5 μM) and Antimycin A (5 μM) for 6 h. Cell lysates were prepared in lysis buffer (50 mM HEPES-HCl pH7.4, 150 mM NaCl, 1 mM EDTA, 10% glycerol, 0.8% Chaps, and 1x HALT phosphatase and protease inhibitor cocktail). After incubating cell lysates on ice for 15 min, they were centrifuged at 20,000 rcf for 15 min. Protein concentration was measured using the Pierce BCA protein assay kit (ThermoFisher, 23227). The appropriate amount of protein lysates was then incubated with antibody-bound Dynabeads^®^ Protein G (ThermoFisher, 10004D) with end-over-end rotation for 3 h at 4 °C. Magnetic beads were washed four times with lysis buffer and transferred to a clean tube for one additional wash. Co-immunoprecipitated protein complexes were eluted by boiling in 2x Laemmli buffer plus β-mercaptoethanol for 5 min. For Western blot analysis, cells in 6-well plates with the indicated treatment were lysed in Triton X-100 lysis buffer (25 mM Tris-HCL pH7.4, 150 mM NaCl, 10% glycerol, 1% Triton X-100 with 1x HALT phosphatase and protease inhibitor cocktail). The lysate concentration was measured using the Pierce BCA protein assay kit. Approximately 25~50 μg protein per sample was separated on SDS-PAGE and wet transferred to 0.2 µm pore size nitrocellulose membranes (Bio-Rad, 1620112) in Tris-glycine transfer buffer. Detection of target proteins in blots was performed using HRP-conjugated secondary antibodies and ECL chemiluminescent substrate (ThermoFisher, PI32106). The following primary antibodies were utilized in Western blotting: rabbit anti-LC3A/B (12741S, Cell Signaling Technology, 1:1000), mouse anti-Flag M5 (F4042, Sigma, 1:4000), rabbit anti-Myc (2278S, Cell Signaling Technology, Danvers, MA, USA, 1:1000), mouse anti-Parkin (4211S, Cell Signaling Technology, 1:1000), rabbit anti-PINK1 (6946S, Cell Signaling Technology, 1:500), rabbit anti-GFP (A11122, ThermoFisher, 1:1000), mouse anti-β-Actin (3700S, Cell Signaling Technology, 1:1000), mouse anti-α-Tubulin (T6074, Sigma, 1:3000), mouse anti-GAPDH(sc-47724, Santa Cruz, 1:1000). Additionally, rabbit anti-GRAF1 and rabbit anti-GRAF1 phospho-S668, rabbit anti-phosphor-S671 polyclonal antibodies were homemade antibodies developed in our lab.

### 2.6. PIP Strips—Lipid-Protein Interaction Assay

PIP Strip (Echelon Biosciences, Salt Lake City, UT, USA, P-6001) was blocked in blocking buffer in TBST with 1% non-fat milk at room temperature for 1 h with gentle agitation. The blocking buffer was discarded, and 1 μg/mL purified GRAF1 protein in 5 mL blocking buffer was added to cover the membrane. The membrane was incubated for 1 h at room temperature with gentle agitation. The protein solution was discarded, and the membrane was washed with >10 mL TBST three times with gentle agitation for 15 min each time. The wash solution was discarded, and the membrane was incubated with rabbit anti-GRAF1 antibody diluted in blocking buffer at room temperature for 1 h, followed by washing three times with TBST for 15 min each time. The wash solution was discarded. The bound protein was detected using the ECL Western Blotting Substrate.

### 2.7. Anti-Phospho-GRAF1 Antibody Generation

The following peptides, synthesized by Thermo Fisher at >90% purity, were utilized for the generation of anti-phospho-GRAF1 antibodies based on GRAF1b isoform amino acids:

Phospho-S668-KLH: LPPNP[S]PTSPLSPS[C]-KLH

Phospho-S668: LPPNP[S]PTSPLSPS

Phospho-S671-KLH: PNPSPT[S]PLSPSW[C]-KLH

Phospho-S671: PNPSPT[S]PLSPSW

Unmodified Peptide: PNPSPTSPLSPSW

Phosphorylation modification sites are denoted by [S], and the addition of Cysteine at the C-terminus of synthetic peptides, followed by conjugation to the carrier protein keyhole limpet hemocyanin (KLH), is represented as [C]-KLH. Phospho-peptides conjugated to KLH were sent to Cocalico Biologicals, Inc. to produce a polyclonal antibody, following a standard 91-day protocol in rabbits. The purification of anti-phospho-peptide antibodies involved the use of two peptide columns—a non-phospho-peptide column to eliminate antibodies recognizing total protein and a phospho-peptide column for the affinity purification of phospho-specific antibodies. The purification was performed as described in references [39,40,41].

### 2.8. GRAF1 Phosphorylation in Human Myocardial Tissue

Human myocardial tissue samples obtained from the Duke Human Heart Repository (DHHR), with approval from the Duke University Hospital Institutional Review Board (IRB No. Pro00005621), were used for this study. Samples were collected from male patients aged 30–60, including those with hypertrophic cardiomyopathy (HCM), dilated cardiomyopathy (DCM), and non-failing (NF) controls, with six samples per group. Tissue collection involves informed consent or a waiver of consent for discarded tissues. Left ventricular (LV) cardiac tissue from HCM and DCM patients was acquired during cardiac transplantation, while LV cardiac tissue from NF subjects was obtained from deceased donors. The collected tissue was promptly dissected, flash-frozen using liquid nitrogen, and stored at −80 °C until use. Patient data available at the time of tissue procurement was retrospectively gathered from electronic medical record databases or organ donor records. Heart tissue homogenization was carried out in Triton X-100 lysis buffer, and GRAF1 phosphorylation in the lysate was analyzed through Western blotting, following established protocols.

### 2.9. Metabolite Extraction, Profiling, and Metabolomic Data Analysis

Metabolite extraction and profiling were performed following previously described methods [42]. Ultimate 3000 UHPLC (Dionex, Sunnyvale, California, USA) coupled to Q Exactive Plus-Mass spectrometer (QE-MS, Thermo Scientific) was used for metabolite profiling. A detailed LC method was described previously [43], except that mobile phase A was replaced with water containing 5 mM ammonium acetate (pH 6.8). LC-MS peak extraction and integration were performed using the commercially available software Sieve 2.2. The integrated peak area represented the relative abundance of each metabolite in different samples. The data matrix of metabolites extracted from mouse serum was normalized by median and then auto-scaled (mean-centered and divided by the standard deviation of each variable). Metabolite pathway analysis, hierarchical clustering, heatmap generation, and statistical analysis were conducted using MetaboAnalyst 5.0 (http://www.metaboanalyst.ca/MetaboAnalyst/, (accessed on 1 January 2022)).

### 2.10. Statistical Calculations

Differences were analyzed using a two-tailed unpaired Student’s t-test for experiments with two groups. A *p*-value < 0.05 was considered statistically significant. Sample sizes were not statistically predetermined. Data are presented as mean ± SD from at least three independent experiments or at least three biological samples. All statistical analyses were performed using GraphPad Prism 10.

## 3. Results

### 3.1. GRAF1 Facilitates Mitochondrial Clearance in Cardiomyocytes

We recently showed that GRAF1 promotes the release of damaged mitochondria from F-actin anchors, regulates mitochondrial-associated Arp2/3-mediated actin remodeling, and facilitates Parkin-LC3 interactions to enhance mitochondria capture by autophagosomes [39]. Our initial insight into GRAF1′s function largely centered on its ability to serve as a RhoA GAP to regulate actin remodeling-dependent mitophagy in Hela cells. To confirm that GRAF1 functions similarly in cardiomyocytes, we subjected neonatal rat ventricle cardiomyocytes (NRVCMs) to treatment with mitochondrial toxins, oligomycin, and antimycin A (OA). 

**Figure 1:**
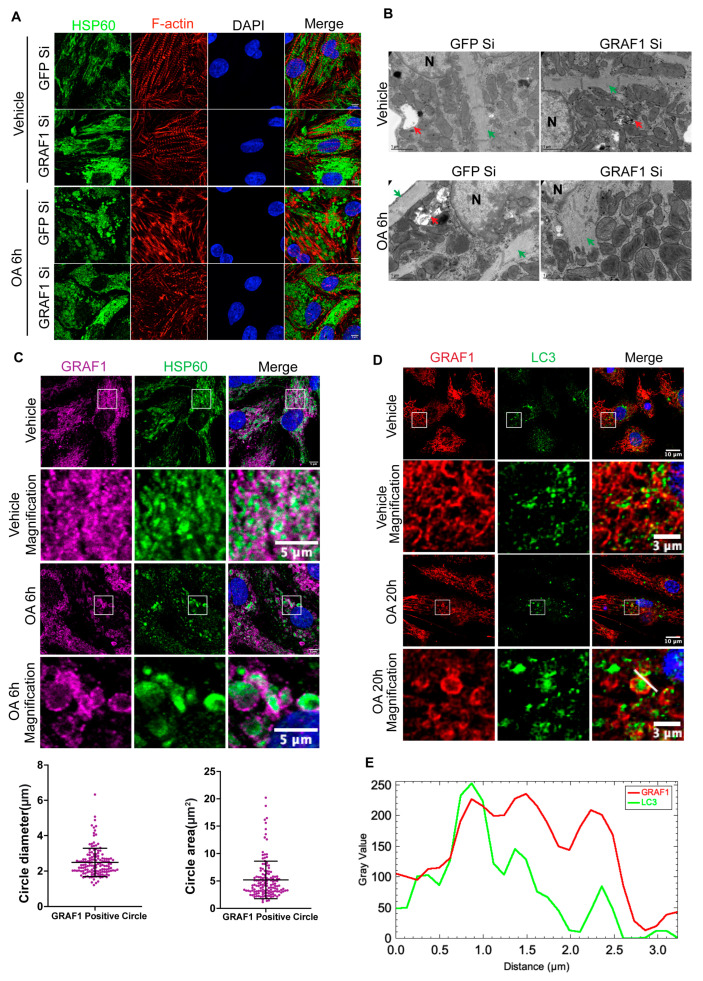
GRAF1 is required for mitochondria clearance in cardiomyocytes. (**A**) Representative confocal images of actin filaments (F-actin) and mitochondria (stained with anti-HSP60 Ab) in NRVCMs. NRVCMs were transfected by indicated siRNA for 96 h, followed by Oligomycin (5 μM) and Antimycin A (5 μM) treatment for 6 h. Note actin remodeling and mitochondrial aggregation following OA treatment. Scale bar: 5 μm. (**B**) Representative TEM of NRVCMs treated as in panel (**A**). N denotes cell nucleus. Red arrows indicate autophagosomes. Green arrows indicate sarcomere structure. (**C**) Representative confocal images of endogenous GRAF1 and mitochondria in NRVCMs that were treated with Vehicle or OA for 6 h. GRAF1 labels ring-like structures in OA condition. Scale bar: 5 μm (Top panel). Quantification of GRAF1 labeled ring structure diameter and area in OA treated NRVCMs (Bottom panel). (**D**) Representative confocal images of endogenous GRAF1 and LC3 in NRVCMs that were treated with Vehicle or OA for 20 h. Scale bars: 10 μm or 3 μm (magnified images). Note that large LC3 puncta are located within some GRAF1-labeled ring structures, while small LC3 puncta are present in the GRAF1-labeled ring structures. (**E**) The intensity profile of endogenous GRAF1- and LC3-positive puncta along the white line that is indicated in the OA 20 h magnified image in (**D**).

Notably, upon exposure to OA, we observed a distinct rearrangement of actin filaments, characterized by the formation of branched actin structures associated with mitochondria labeled by staining for the mitochondria matrix protein HSP60 (Figure 1A). In addition, OA treatment also induced the formation of large circular clusters of mitochondria in close proximity to the cell nucleus in OA-exposed NRVCMs transfected with GFP siRNA. However, the actin remodeling, mitochondrial circularization, and clustering induced by OA were significantly reduced in cardiomyocytes lacking GRAF1, highlighting the crucial role of GRAF1 in these processes (Figure 1A). TEM analysis of similarly treated NRVCMs supported our findings from confocal images. In control conditions, sarcomeres were well-developed, and mitochondria exhibited a dispersed distribution, occupying a significant portion of the cytoplasmic volume. Contrastingly, OA treatment led to sarcomere disassembly, actin remodeling, and clusters of mitochondria closely associated with actin that fused with autolysosomes near the cell nucleus in GFP siRNA-transfected NRVCMs. Importantly, GRAF1-depleted NRVCMs showed defects in forming branched structures, and mitochondria still exhibited a relatively dispersed pattern within the cytoplasm (Figure 1B). Moreover, we observed that OA exposure induced the formation of circularized structures of endogenous GRAF1 surrounding the mitochondria in NRVCMs (Figure 1C, top panel). These GRAF1-positive ring structures exhibited an average diameter of 2.49 μm ± 0.79 and an average area of 5.19 μm^2^ ± 3.42 (Figure 1C, bottom panel), similar to the size of autophagosomes previously reported to capture large cargo like mitochondria [44]. Furthermore, we observed that these GRAF1-labeled ring structures were co-labeled with endogenous LC3 (Figure 1D,E), a reliable marker for autophagosome [45]. These findings suggest that in addition to the regulation of mitochondrial-associated actin, GRAF1 may play an important role in the targeted removal of damaged mitochondria by promoting their selective engulfment into the autophagosome.

### 3.2. GRAF1 Associates with LC3

**Figure 2:**
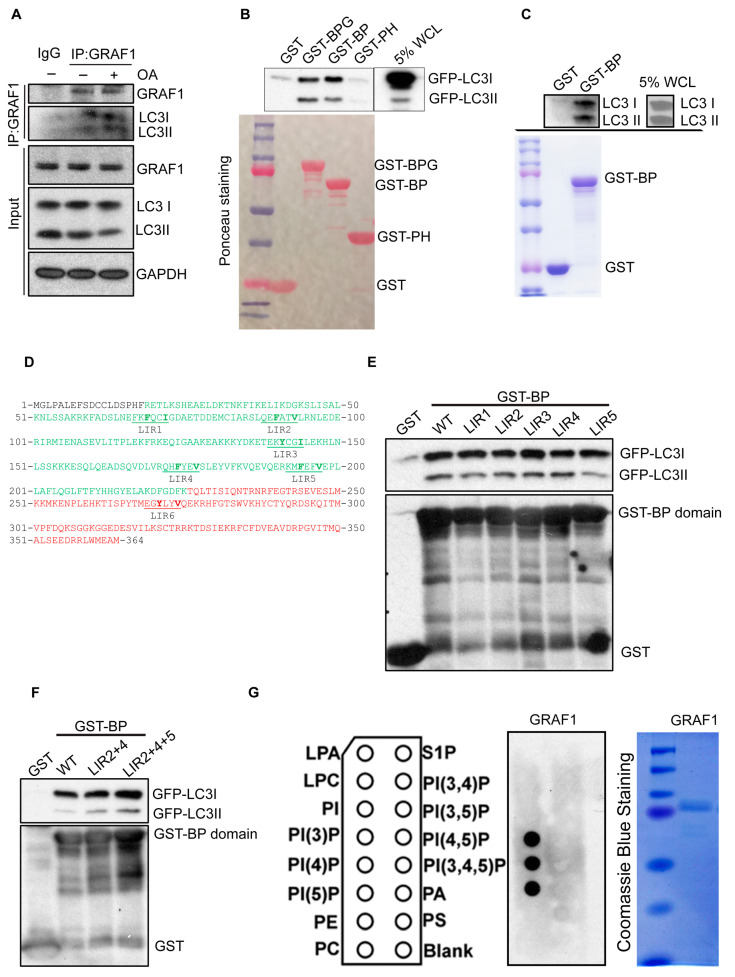
GRAF1 binds to PI(3)P, PI(4)P, and PI(5)P, forming a complex with LC3. (**A**) Co-IP of endogenous LC3I/II with endogenous GRAF1 in NRVCMs treated with OA treatment for 6 h. (**B**) In vitro interaction of purified GST-GRAF1 domains with exogenously expressed GFP-LC3I/II. Bottom: Ponceau S staining of purified GST-fusion protein input. BPG: GRAF1 BAR-PH-GAP domains. BP: GRAF1 BAR-PH domains. WCL: whole cell lysate. (**C**) In vitro interaction of purified GST-GRAF1 BAR and PH domains with endogenous LC3I/II in NRVCMs. Bottom: Coomassie blue staining of purified GST-fusion protein input. BP: GRAF1 BAR-PH domains. (**D**) Amino acid sequence of GRAF1 BAR and PH domains. The BAR domain sequence is highlighted in green, and the PH domain sequence is highlighted in red. The putative LIR motif(1-6) sequence is indicated by the underscore line. (**E**,**F**) In vitro interaction of purified GST-GRAF1 BAR and PH domains containing individual putative LIR mutant (**E**) or LIR mutant combination (**F**) with exogenously expressed GFP-LC3I/II. Bottom: purified GST-fusion protein input was assessed by western blot. (**G**) Lipid and protein interaction was assessed by PIP strip. Left: Schematic of PIP strip showing individual lipid spots on the membrane. Middle: Purified GRAF1 binding to lipid spot on the membrane was detected by HRP labeled antibody and ECL. Right: Coomassie blue staining of purified GRAF1 used in Lipid binding assay.

LC3 is a ubiquitin-like protein that, upon activation, undergoes a covalent linkage to phosphatidylethanolamine (PE) on the autophagosome membrane. The association of LC3 to autophagosomes promotes isolation membrane expansion and participates in the recruitment of cargo into the autophagosome[46]. Cargo recruitment is largely accomplished by protein-protein interactions driven by LC3-interacting region (LIR) motifs within LC3 binding adapter proteins [23,24,25]. To begin to understand how GRAF1 might mediate this process to facilitate the autophagosomal capture of damaged mitochondria, we first investigated if GRAF1 might function by interacting directly with LC3. Notably, we found that endogenous GRAF1 co-immunoprecipitated with endogenous LC3 I/II in NRVCMs (Figure 2A).

Importantly, this interaction was enhanced following treatment with OA, implying that GRAF1 may form a complex with LC3 to facilitate the targeting of autophagosomes to damaged mitochondria. Subsequently, we sought to identify the specific domain of GRAF1 responsible for mediating this interaction using a GST fusion protein-pulldown assay. Our findings revealed that the GRAF1 GST fusion protein containing both the BAR and PH domains robustly precipitated GFP-LC3 I/II (Figure 2B). Furthermore, we demonstrated that purified GST-GRAF1 BAR-PH domains efficiently pulled down endogenous LC3 I/II from NRVCMs, indicating that the BAR-PH domains serve as mediators in the interaction with LC3 (Figure 2C).

The canonical LIR motif is characterized by a short amino acid sequence with a core motif originally described as **W/F/Y**xx**I/L/V** (where ‘x’ represents any amino acids). Recently, this sequence has been expanded to six amino acids, forming the new consensus sequence [ADEFGLPRSK][DEGMSTV][**WFY**][DEILQTV][ADEFHIKLMPSTV][**ILV**] [47]. The residues in positions 3 and 6 are particularly crucial for interaction with Atg8-family proteins. Utilizing the iLIR database (https://ilir.warwick.ac.uk (accessed on 1 January 2020), we identified six putative LIR motifs within the GRAF1 BAR-PH domains (19-364 amino acids), with five located within the BAR domain and one within the PH domain (LIR1-6) (Figure 2D). To determine if these motifs were essential for LC3 interaction, we mutated the key amino acids at positions 3 and 6 in each putative LIR motif to alanine. Somewhat surprisingly, GST fusion protein pulldown assays revealed that neither single LIR mutations nor combinations of multiple LIR mutations could abolish the GRAF1-LC3 interaction (Figure 2E,F). This suggests that GRAF1′s interaction with LC3 is not likely mediated by the putative LIR motifs in the BAR-PH domains.

Given that the BAR-PH domains inherently function to bind lipid membranes and modulate membrane curvature and that LC3 is conjugated to autophagosome membranes, we next hypothesized that the observed GRAF1-LC3 complex may result from GRAF1’s ability to recognize and bind autophagosome membranes. To investigate this further, we expressed and purified GRAF1 and assessed its binding capacity and specificity to various lipids by a lipid-protein interaction assay. Remarkably, GRAF1 exhibited high specificity and affinity for PI(3)P, PI(4)P, and PI(5)P (Figure 2G). These phospholipids play crucial roles in autophagosome biogenesis, membrane expansion, and fusion with lysosomes by interacting with their distinct effector proteins [48,49,50]. Taken together, our findings suggest that GRAF1 associates with LC3 by binding to PI(3)P, PI(4)P, and PI(5)P on autophagosomes. When combined with our prior findings that the formation of circularized membrane structures encasing damaged mitochondria requires GRAF1 [39], these data suggest that GRAF1 may play a role in autophagosome closure, possibly through BAR-mediated membrane bending [38,51].

### 3.3. GRAF1 Facilitates PINK1-Parkin Dependent Recruitment of Autophagosomes to Damaged Mitochondria

**Figure 3:**
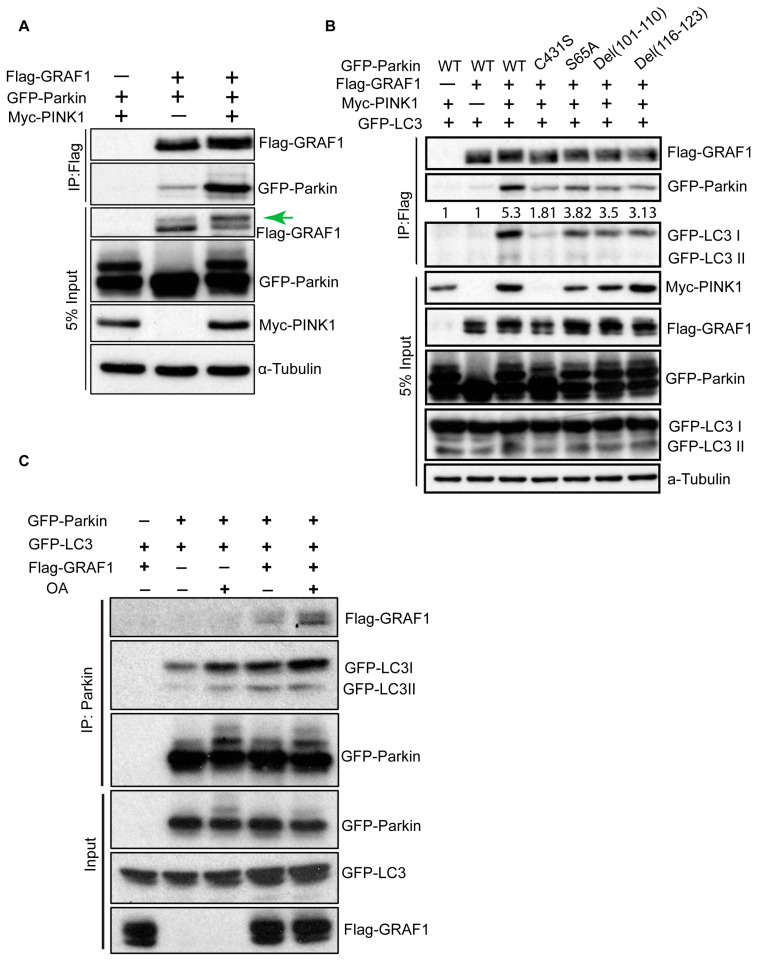
GRAF1 forms a complex with Parkin and LC3. (**A**) Co-IP of GFP-Parkin in COS7 cells co-transfected with Flag-GRAF1, along with either Myc-PINK1 or empty vector. The green arrow indicates the GRAF1 band shift on SDS-PAGE gel. (**B**) Co-IP of GFP-Parkin variants and GFP-LC3I/II with Flag-GRAF1 in COS7 cells co-transfected with Myc-PINK1. The densitometric intensity value of GFP-LC3 was indicated. (**C**) Reciprocal Co-IP of Flag-GRAF1 and GFP-LC3I/II with GFP-Parkin in co-transfected COS7 cells treated with Vehicle or OA (2.5 μM Oligomycin, 250 nM Antimycin A) for 6 h.

The PINK1-Parkin pathway plays a crucial role in promoting mitophagy by ubiquitinating proteins on the mitochondrial outer membrane (MOM). This initiates the recruitment of ubiquitin-binding adapter proteins, which subsequently interact with LC3, facilitating the assembly of autophagosomal membranes around ubiquitylated mitochondria. Given the significance of GRAF1 in the clearance of damaged mitochondria and its association with LC3, our subsequent investigation sought to determine whether GRAF1 acts as a mediator in the PINK1-Parkin signaling pathway. We found that GRAF1 forms a complex with Parkin, and this interaction is regulated by PINK1. This is evident from the enhanced GRAF1-Parkin interaction with PINK1 co-expression in co-immunoprecipitation (Figure 3A). When taken together with our prior findings that GRAF1 associates with fully active Parkin [39], these data collectively indicate that GRAF1 can facilitate the selective engulfment of damaged mitochondria by directly associating with Parkin and indirectly associating with LC3 via binding to autophagosome phospholipids.

Intriguingly, under the PINK1 co-expression condition, GRAF1 exhibited a mobility shift that was apparent by SDS-PAGE, indicative of a potential post-translational modification. Parkin is an E3 ubiquitin ligase that exists in an autoinhibitory state at basal levels. Its activity is modulated by PINK1-mediated phosphorylation at S65 and the conserved regions in the linker between the ubiquitin-like (Ubl) and RING0 domains [52]. GRAF1 Co-IP revealed that GRAF1 also forms a complex with Parkin and LC3, and their interaction is mediated by PINK1 and Parkin activity, as evidenced by induced complex formation upon Parkin activation through PINK1 expression. Conversely, reduced Parkin-GRAF1-LC3 association was observed with Parkin harboring a ubiquitination-defective mutation (C431S), S65A non-phosphorylatable variant, or deletion of conserved regions in the linker (Figure 3B). Moreover, reciprocal Parkin Co-IP experiments validated that OA-induced mitophagy enhances Parkin-GRAF1-LC3 complex formation and that GRAF1 promotes the association between Parkin and LC3 (Figure 3C). These findings provide strong support for the notion that GRAF1 serves as a downstream mediator in the PINK1-Parkin pathway to facilitate the recruitment of autophagosomes to damaged mitochondria.

### 3.4. GRAF1 Phosphorylation Is Mediated by the PINK1-Parkin Pathway

**Figure 4:**
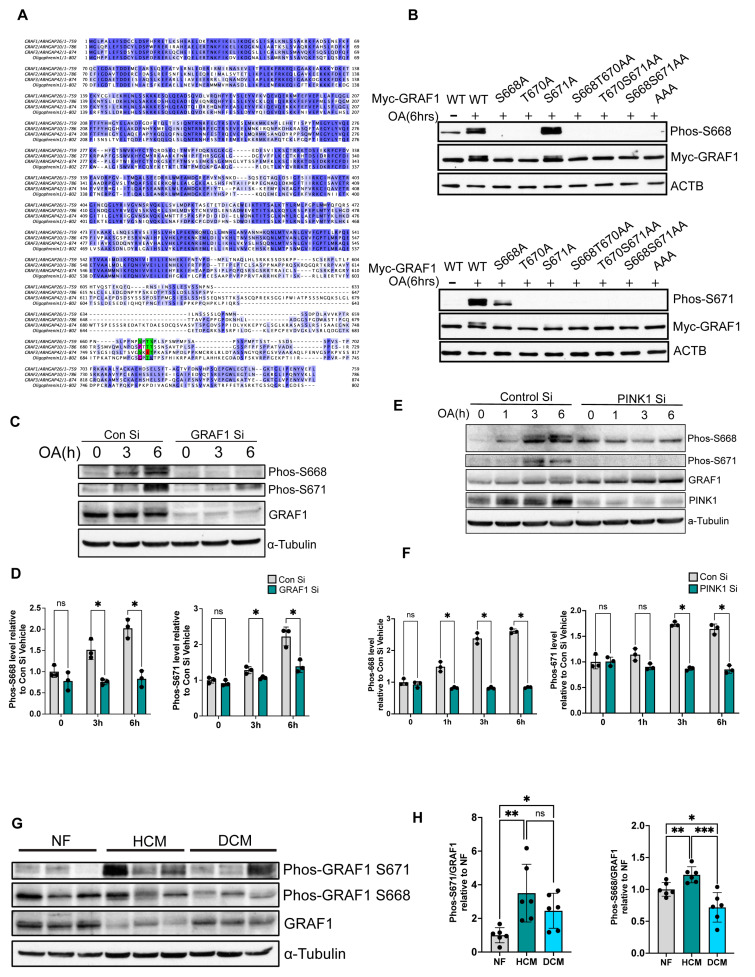
GRAF1 phosphorylation at S668 and S671 is mediated by PINK1. (**A**) Amino acid sequence alignment of indicated GRAF family members. The positions of S668, T670, and S671 in human GRAF1b are highlighted to show conservation across family members. Green denotes conserved S668/T670/S671 sites within GRAF1 family members, while red indicates divergent S668/T670/S671 sites within the family. (**B**) Validation of rabbit anti-GRAF1 phos-S668 and rabbit anti-GRAF1 S671 antibodies by western blot. Myc-GRAF1 WT and indicated mutants were transfected in Hela/YFP-Parkin cells for 20 h, followed by OA treatment for 6 h. (**C**,**D**) Validation of rabbit anti-GRAF1 phos-S668 and rabbit anti-GRAF1 S671 antibodies by western blot using Hela/YFP-Parkin cells transfected with specified siRNA for 72 h, followed by OA treatment for the indicated time and quantified by densitometry (**D**, *n* = 3)). (**E**,**F**) GRAF1 phosphorylation was examined by Phos-S668 and Phos-S671 antibodies in Hela/YFP-Parkin cells transfected with specified siRNA for 72 h followed by OA treatment and quantified by western blot/densitometry (**F**, *n* = 3)). (**G**,**H**) GRAF1 phosphorylation at S668 and T671 was assessed in human heart samples by western blot (**G**) and densitometric quantification (**H**). *n*  =  6/group. NF: none failing, HCM: hypertrophic cardiomyopathy. DCM: dilated cardiomyopathy. Data in (**D**,**F**,**H**) are represented as mean ± SD. ns, not significant; * *p*  <  0.05, ** *p*  <  0.01, *** *p*  <  0.001 by two-tailed student’s *t*-test.

As noted above, we observed a distinct GRAF1 band shift following OA treatment, suggesting a post-translational modification (Figure 3A, green arrow). Our previous study identified S668, T670, and S671 as the major GRAF1 phosphorylation sites mediated by the PINK1-Parkin pathway [39]. Notably, these amino acids exhibit partial conservation in other GRAF family members. For instance, human GRAF2 contains T670 and S671, human GRAF3 contains S668 and S671, and human Oligophrenin1 contains T670 (Figure 4A).

We previously made and characterized an antibody that selectively recognized GRAF1 phosphorylation at all 3 identified PINK1-dependent phosphosites [39]. To begin to characterize the regulation and consequence of individual phosphorylation events, we next sought to develop phospho-antibodies that selectively detect phosphorylation of GRAF1 S668 or S671. As shown in Figure 4B, we confirmed that OA-induced GRAF1 phosphorylation at these sites by analyzing various GRAF1 mutational variants (Figure 4B). Interestingly, mutation of T670 to a phosphorylation-deficient A670 attenuated OA-mediated phosphorylation of S668 and S671, suggesting that T670 phosphorylation is a prerequisite for subsequent phosphorylation at S668 and S671 (Figure 4B). Using these novel phosphor-specific antibodies, we found that phosphorylation of endogenous GRAF1 at S668 and S671 peaked at 6 h following OA treatment. Notably, we further confirmed the specificity of the antibodies to detect endogenously phosphorylated GRAF1 in Hela cells. As shown in Figure 4C,D, siRNA-mediated GRAF1-depletion fully attenuated phosphor-GRAF1 S668 and S671 detection (Figure 4C,D). Given that GRAF1 phosphorylation occurs in OA-treated cells and PINK1 accumulates on the outer membrane of damaged mitochondria, we explored the possibility that GRAF1 phosphorylation of these two sites is mediated by PINK1. We found that depletion of PINK1 reduced OA-stimulated phosphorylation of endogenous GRAF1 at both S668 and S671, as indicated by the intensity of detection with Phospho-S668 and Phospho-S671 antibodies (Figure 4E,F). This finding strongly supports the notion that GRAF1 phosphorylation is mediated by PINK1 itself and/or additional PINK1/Parkin-dependent kinases.

Interestingly, our investigation of cardiac samples from patients diagnosed with hypertrophic or dilated cardiomyopathy (HCM or DCM) revealed distinct dysregulation in GRAF1 S668 and T671 phosphorylation compared to controls matched for age and sex (Figure 4G). Notably, compared to normal samples, S671 phosphorylation is significantly upregulated in both hypertrophic cardiomyopathy (HCM) and dilated cardiomyopathy (DCM) samples, while S668 is significantly upregulated in HCM and significantly reduced in DCM samples(Figure 4H). Thus, while phosphorylation of both sites is PINK1-dependent, different phosphatases may regulate the de-phosphorylation of these sites. These collective findings suggest that dysregulation of GRAF1 phosphorylation may contribute to the exacerbation of cardiac diseases and potentially other conditions associated with defects in PINK1/Parkin-mediated mitochondrial quality control.

### 3.5. GRAF1 Is Required for Stress-Induced Metabolic Flexibility in the Heart

In our previous study, we observed compromised cardiac mitochondrial fitness in the hearts of GRAF1^gt/gt^ mice subjected to ISO treatment, a β-adrenergic agonist increasing cardiac energy demand [39]. We also established a tamoxifen-inducible mouse model with cardiomyocyte-specific GRAF1 knockout (GRAF1^CKO^) to investigate the autonomous role of GRAF1 in cardiomyocytes for maintaining mitochondrial homeostasis. Our study demonstrated that adult GRAF1^CKO^ mice maintained normal cardiac physiology following acute GRAF1 depletion; however, exposure to ISO induced cardiac dysfunction in these mice, which, at least in part, is attributed to variations in cardiac substrate metabolism.

**Figure 5:**
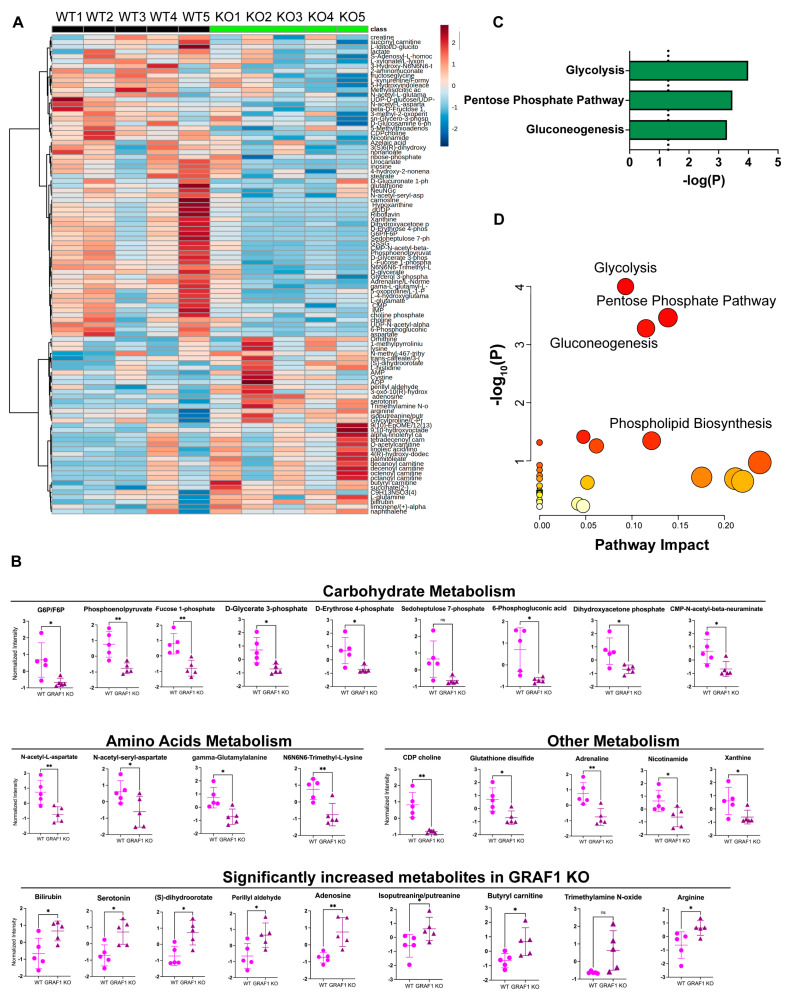
ISO−treated WT and GRAF1^CKO^ mice exhibit distinct metabolic profiles in serum. (**A**) Supervised hierarchical clustering heatmap showing the top 100 different metabolites in serum from ISO−challenged WT and GRAF1^CKO^ mice. *n*=5 mice/group. (**B**) Relative normalized intensity levels of representative carbohydrates, amino acid derivatives, and some other metabolites exhibited changes in abundance in the serum from ISO-treated WT and GRAF1^CKO^ mice. (**C**) Histogram showing significantly upregulated metabolic pathways (*p* < 0.05) resulting from statistically differed metabolites shown in (**B**). (**D**) The significantly different and most impacted pathways were identified in serum from ISO-treated WT and GRAF1^CKO^ mice through pathway analysis. ns, not significant; * *p*  <  0.05, ** *p*  <  0.01 by two-tailed student’s *t*-test.

Cardiomyocyte mitochondria have the capacity to utilize various energy substrates (carbohydrates, lipids, amino acids, and ketone bodies) for ATP production, rendering the cardiac metabolic network highly flexible in response to stimuli [53,54,55]. To investigate whether altered cardiac metabolism resulting from GRAF1 knockout in the heart also leads to alterations in circulating metabolites, we subjected serum collected from ISO-treated WT and GRAF1^CKO^ mice to unbiased metabolomics approaches. This involved using liquid chromatography coupled with high-resolution mass spectrometry (LC-HRMS) to analyze the metabolite profiles. Notably, the metabolites from ISO-treated WT and GRAF1^CKO^ serum clustered into two distinct groups, indicating that the serum metabolic profile was robustly influenced by GRAF1 knockout in the heart (Figure 5A).

As shown in Figure 5B, the most significantly enriched metabolites in WT serum were intermediates of carbohydrate metabolism, along with a few amino acid-derived metabolites related to phospholipid biosynthesis. Subsequent pathway enrichment and topology analysis of these significantly different metabolites identified glycolysis, pentose phosphate pathway, and gluconeogenesis as the pathways significantly altered between the two genotypes (Figure 5C,D). This is consistent with prior reports that cardiac stress promotes a shift in fuel utilization from predominantly fatty acids to other available substrates like glucose and amino acids [56,57]. Collectively, our findings from the assessment of serum metabolites indicate that GRAF1^CKO^ hearts exhibit impaired metabolic flexibility, specifically in efficiently utilizing carbohydrates as an alternative substrate for energy production. This observation aligns with our previous study on GRAF1^CKO^ heart tissue metabolism and with the emerging idea that mitochondrial quality control mechanisms, including mitophagy, are essential to match energy demands with fuel supply.

## 4. Discussion

Cardiomyocytes have the highest mitochondrial density of all cells, and they depend on these healthy mitochondria for the continuous generation of ATP to sustain their contraction [58,59]. Disruption of mitochondria quality control has been implicated in heart diseases, including myocardial infarction, heart failure, and dilated cardiomyopathy [60,61,62,63]. Therefore, unraveling the intricate regulatory mechanisms underlying mitochondria quality control in cardiomyocytes will not only enhance our understanding of mitochondria-related pathophysiological processes in these cells but will also prove beneficial in the future development of effective treatment options for certain cardiac diseases.

Here, we show that GRAF1 functions as a downstream mediator of PINK1-Parkin to facilitate mitophagy by linking Parkin-labeled mitochondria and LC3-labeled autophagosomes. Upon exposure to mitochondria toxins, GRAF1 is rapidly redistributed to ring-like structures that envelop mitochondria. Interestingly, LC3 puncta coexist within these GRAF1-positive ring structures, suggesting a potential complex formation between GRAF1 and LC3. Subsequent endogenous GRAF1 Co-IP in NRVCMs revealed a direct interaction with LC3, particularly in response to mitophagy induction by mitochondrial poisons.

To explore the molecular basis of this interaction, we employed a GST fusion protein pulldown assay, which uncovered a role for GRAF1’s BAR-PH domains in mediating the interaction with LC3. Typically, protein receptors interact with LC3 through LC3-interacting regions (LIRs) [23,24,25]. Leveraging an online database (https://ilir.warwick.ac.uk, (accessed on 1 January 2020)) [47], we identified a total of six putative LIR motifs in human GRAF1 BAR-PH domains, with five within the BAR domain and one in the PH domain. Significantly, five out of the six LIRs are evolutionarily conserved in GRAF1 from frogs to humans. However, our in vitro GST-fusion protein pulldown assay revealed that neither individual mutation nor a combination of these LIR motifs reduces LC3 binding. This suggests that BAR-PH domain-mediated LC3 interaction is not regulated by LIR motifs but rather by other mechanisms.

The BAR domain forms an elongated banana-shaped homodimer that is crucial for binding membranes as well as sensing and inducing membrane curvature through its concave shape [64,65]. The PH domain modulates membrane-binding specificity, directing the protein to a specific membrane compartment enriched in cognate phosphoinositides (PIs) [66,67,68,69]. A recent study revealed that Atg8-containing autophagosome membranes in yeast are comprised of 38% PC, 37% PI, 19% PE, 3% PS, 3% PA, and an abundance of unsaturated phospholipids [70,71]. In light of the covalent conjugation of LC3 to the autophagosome membrane and the collaborative action of GRAF1’s BAR and PH domains in membrane binding, we propose that GRAF1’s interaction with LC3 occurs through its BAR-PH domain binding to the autophagosome membrane. Our strip lipid-protein binding assay demonstrated that purified full-length GRAF1 protein exhibits a high affinity for PI(3)P, PI(4)P, and PI(5)P. These phospholipids are important components of the autophagosomal membrane and play a crucial role in autophagosome biogenesis, expansion, and fusion with lysosomes by interacting with their distinct effector proteins [48,49,50,70]. Taken together, these findings suggest that GRAF1’s BAR and PH domains enable it to co-select the lipid composition and curvature of the autophagosome, ultimately leading to the formation of the GRAF1-LC3 complex.

Notably, our previous study revealed that GRAF1 depletion significantly reduced LC3 colocalization with mitochondria in OA-treated cells and diminished Parkin and GFP-LC3 complex formation [39]. Our current study extends these observations by demonstrating that Parkin forms a complex with GRAF1 and LC3. Moreover, exogenous expression of GRAF1 increases Parkin and LC3 interaction under basal conditions or following OA treatment. These findings collectively support the role of GRAF1 as a mediator in the PINK1-Parkin pathway by serving to guide the autophagosome membrane spatially and temporally to damaged mitochondria. Considering the functional significance of the BAR-PH domains and the characteristics of autophagosome membranes, further investigation into the role of GRAF1 in autophagosome biogenesis and maturation holds significant promise for future studies.

PINK1-Parkin-mediated mitophagy is a crucial mechanism for maintaining mitochondrial quality control [22,72]. Accumulating evidence in cardiac studies suggests that, under basal conditions, the involvement of PINK1-Parkin in mitophagy is limited, but it becomes significantly pronounced during stress conditions such as myocardial infarction or disruption of mitochondrial fission due to Drp1 deletion in cardiomyocytes [73,74,75,76]. Our prior research revealed that GRAF1 undergoes phosphorylation at the S668, T670, and S671 sites within the proline-rich region in stressed cardiomyocytes. Importantly, this phosphorylation is mediated by the active PINK1-Parkin pathway. Under control conditions, GRAF1 phosphorylation at the S668, T670, and S671 sites is low. However, it undergoes a significant upregulation in response to stress [39]. This observation aligns with the dynamic behavior of PINK1-Parkin in the heart.

In the present study, we developed antibodies specifically targeting phosphor-S668 and phosphor-S671 and validated their specificity in GRAF1-depleted cells. Our findings revealed that phosphorylation of S668 and S671 are both mediated by PINK1, but phosphorylation of these sites is dependent on prior T670 phosphorylation. It will be of future interest to determine the kinase responsible for T670 phosphorylation and to test the intriguing possibility that phosphorylation of this site promotes GRAF1 targeting to damaged mitochondria wherein it becomes subsequently phosphorylated by PINK1 on S668 and S671.

Our further characterization herein of these phosphorylation events in human heart samples yielded intriguing findings. Compared to normal samples, S671 phosphorylation was significantly upregulated in both hypertrophic cardiomyopathy (HCM) and dilated cardiomyopathy (DCM) samples. However, S668 was significantly upregulated in HCM and significantly reduced in DCM samples. This suggests that GRAF1 exhibits distinct phosphorylation statuses: in a healthy heart, GRAF1 displays relatively low phosphorylation at these three sites; in HCM samples, all three sites are phosphorylated, and in DCM samples, T670S671 are phosphorylated while S668 phosphorylation is suppressed. This distinct phosphorylation may serve as an indicator of GRAF1’s functional levels. Considering that normal, HCM, and DCM represent three distinct cardiac health statuses, the phosphorylation status of these three sites may serve as biomarkers for heart disease pathogenesis, providing valuable insights for cardiac function evaluation.

The heart requires substantial energy to meet its demanding workload, and a healthy heart exhibits metabolic flexibility by utilizing various substrates such as fatty acids, carbohydrates, amino acids, and ketone bodies for ATP production in the mitochondria [53,54,55]. Typically, under normal conditions, the healthy adult heart primarily relies on fatty acid oxidation for energy generation. However, during acute transitions in workload, there is a shift from fatty acid to glucose oxidation to maintain energy stability [77,78,79]. In our study, the metabolic profile in the serum of ISO-treated GRAF1^CKO^ mice indicates a significantly lower level of carbohydrate-related metabolites. This suggests an impaired transition to using carbohydrates as a substrate to meet increased energy demands during elevated cardiac workload. These findings highlight that GRAF1 depletion disrupts mitochondrial homeostasis, thereby limiting metabolic flexibility in vivo. Ultimately, this limitation adversely affects cardiac functions, as demonstrated by echocardiography in our previous study [39].

In summary, autophagy and selective autophagy is a highly dynamic process that involves the initiation, nucleation, and expansion of autophagosome membranes for target sequestration, culminating in the ultimate closure and fusion with the lysosome. This intricate process engages numerous protein complexes, membrane dynamics, and cytoskeletal remodeling. Despite the identification of many proteins playing crucial roles in specific steps, the coordination of membrane dynamics and cytoskeletal remodeling remains elusive. In our prior study, we investigated the role and underlying mechanism of GRAF1 GAP and SH3 domains in mitochondria-associated actin remodeling during mitophagy. Specifically, upon mitochondrial depolarization, the GRAF1 GAP domain suppresses RhoA-dependent F-actin polymerization. This releases mitochondria from the F-actin filament and generates G-actin monomers for nascent polymerization. Simultaneously, phosphorylation at S668/T670/S671 sites releases the autoinhibitory state of the SH3 domain from its upstream proline-rich region, enabling GRAF1 to interact with ABI2 and WAVE2 complex. This interaction leads to branched actin formation that is necessary for efficient mitochondria clearance [39]. Our latest data in the present study shed light on the role of the N-terminal BAR-PH domains in recruiting autophagosome membranes to damaged mitochondria. Together, our findings strongly support the notion that GRAF1 acts as a downstream mediator of Parkin to spatiotemporally coordinate actin and membrane dynamics and facilitate PINK1-Parkin-dependent mitophagy in energetically stressed cardiomyocytes.

## Data Availability

The raw metabolomic data for this study are deposited in the University of North Carolina Digital Repository https://doi.org/10.17615/7x9x-0q90 accessed on 1 January 2024. The scanned blots and metabolic data are deposited in the University of North Carolina Digital Repository with access code 7d279496f.
